# Oncolytic Virotherapy and Immunogenic Cell Death: Mechanisms, Platforms, and Clinical Translation

**DOI:** 10.3390/v18040461

**Published:** 2026-04-13

**Authors:** Hiroyuki Inoue

**Affiliations:** Department of Respiratory Medicine, Fukuoka University Hospital, Fukuoka 814-0180, Japan; hinoue@fukuoka-u.ac.jp; Tel.: +81-092-811-1011

**Keywords:** oncolytic virus, immunogenic cell death, checkpoint inhibitor, coxsackievirus, coxsackievirus A11, type I interferon, tumor microenvironment, cancer immunotherapy

## Abstract

Oncolytic viruses represent a paradigm-shifting approach to cancer immunotherapy, functioning as in situ vaccines that convert immunologically “cold” tumors into “hot” tumors through induction of immunogenic cell death (ICD). Despite the clinical success of checkpoint inhibitors targeting programmed cell death protein 1 (PD-1)/programmed death-ligand 1 (PD-L1) and cytotoxic T-lymphocyte-associated protein 4 (CTLA-4), many patients exhibit primary or acquired resistance due to insufficient tumor immunogenicity and exclusion of tumor-infiltrating lymphocytes. Oncolytic viruses address this limitation by selectively replicating in tumor cells, inducing robust ICD characterized by four cardinal hallmarks: calreticulin exposure, ATP secretion, HMGB1 release, and type I interferon production. This review systematically examines the molecular mechanisms underlying virus-induced ICD, compares DNA virus platforms (Vaccinia, HSV-1, Adenovirus) with RNA virus platforms (Coxsackieviruses A21, A11, and B3), and analyzes clinical trial data demonstrating synergistic efficacy when combined with checkpoint inhibitors. Notably, RNA viruses generate higher type I interferon responses compared to DNA viruses, correlating with superior clinical outcomes. Coxsackievirus A21 combined with pembrolizumab achieved a 47% objective response rate in melanoma in the CAPRA trial, representing notable efficacy exceeding either monotherapy. Coxsackievirus A11 demonstrates exceptional selectivity for thoracic cancers through ICAM-1-dependent receptor tropism and potent immunogenic cell death induction. Japanese researchers have pioneered microRNA-targeted Coxsackievirus B3, achieving cardiac safety attenuation while preserving complete oncolytic potency and ICD-inducing capacity. This comprehensive analysis synthesizes molecular mechanisms, platform comparisons, clinical efficacy data, and translational challenges to guide future development of oncolytic virotherapy as a cornerstone of cancer immunotherapy.

## 1. Introduction: The Challenge of Immunologically Cold Tumors

### 1.1. Checkpoint Inhibitors and the Cold Tumor Problem

The advent of immune checkpoint inhibitors targeting PD-1/PD-L1 and CTLA-4 has revolutionized cancer therapy, demonstrating durable responses and long-term survival benefits across multiple cancer types [1,2]. Ipilimumab, the first FDA-approved CTLA-4 inhibitor, improved overall survival in metastatic melanoma patients, establishing immunotherapy as a viable treatment modality [3]. Subsequently, PD-1/PD-L1 inhibitors have achieved approval for melanoma, non-small cell lung cancer, renal cell carcinoma, bladder cancer, and numerous other malignancies, with some patients achieving complete responses lasting years beyond treatment cessation [1,2]. However, the majority of cancer patients fail to respond to checkpoint inhibitor monotherapy. Response rates typically range from 15–45% depending on tumor type, with primary resistance occurring in 40–65% of patients and acquired resistance developing in 25–40% of initial responders [4,5]. The fundamental limitation is that checkpoint inhibitors require pre-existing anti-tumor immunity to be effective—they release the “brakes” on T cell responses but cannot initiate de novo immune responses in immunologically “cold” tumors [6,7].

Cold tumors are characterized by absent or minimal CD8+ T cell infiltration, lack of inflammatory gene signatures, and immunosuppressive microenvironments dominated by regulatory T cells, myeloid-derived suppressor cells, and tumor-associated macrophages [8,9]. Multiple mechanisms contribute to immune exclusion, including oncogenic signaling pathways (β-catenin, PTEN loss), stromal barriers (dense collagen deposition, cancer-associated fibroblasts), and immunosuppressive cytokines (TGF-β, IL-10) [7,10]. Tumors with high baseline CD8+ T cell infiltration and PD-L1 expression respond significantly better to checkpoint blockade, with response rates approaching 60–80% in PD-L1-high, CD8+ T cell-inflamed tumors versus <10% in PD-L1-negative, T cell-excluded tumors [11]. The critical unmet need is to transform cold tumors into hot tumors—converting immunologically ignored malignancies into inflamed lesions rich in tumor-infiltrating lymphocytes and responsive to checkpoint inhibition therapies.

### 1.2. Oncolytic Viruses as In Situ Cancer Vaccines

Oncolytic viruses (OVs) represent a fundamentally distinct class of cancer immunotherapy that addresses the cold tumor problem by functioning as in situ vaccines [12,13,14,15,16,17,18,19]. These engineered or naturally occurring viruses selectively replicate in tumor cells due to defects in antiviral defenses common to malignant cells, including impaired interferon signaling, dysfunctional p53 and Rb pathways, and aberrant rat sarcoma (RAS)/mitogen-activated protein kinase (MAPK) activation [12,14]. Selective replication generates 1000–10,000 progeny virions per infected cell over 24–72 h, causing direct tumor cell lysis and spreading infection throughout the tumor mass [13,15].

The therapeutic potential of oncolytic viruses extends far beyond direct cytotoxicity. Virus-mediated tumor cell death induces immunogenic cell death (ICD), a specialized form of cell demise characterized by the spatiotemporally coordinated release of damage-associated molecular patterns (DAMPs) and pathogen-associated molecular patterns (PAMPs) that convert the tumor into an endogenous vaccine [20,21,22]. Talimogene laherparepvec (T-VEC), an engineered herpes simplex virus type 1 (HSV-1) encoding granulocyte-macrophage colony-stimulating factor (GM-CSF), became the first FDA-approved oncolytic virus in 2015 for advanced melanoma treatment, demonstrating a 16.3% durable response rate and significant improvement in tumor-specific CD8+ T cell responses [23,24]. The true promise of oncolytic virotherapy lies in rational combination with checkpoint inhibitors. Preclinical studies demonstrated that intratumoral T-VEC administration overcomes systemic resistance to CTLA-4 and PD-1 blockade, converting cold tumors into hot tumors with dense T cell infiltration [25,26]. This mechanistic synergy—OVs generating new tumor-associated antigens and inflammatory signals such as DAMPs and PAMPs and checkpoint inhibitors releasing T cell re-invigoration—has translated into striking clinical efficacy, with combination regimens achieving response rates 1.7–2.4-fold higher than either monotherapy [25,26,27,28,29].

## 2. Molecular Mechanisms of Immunogenic Cell Death Induced by Oncolytic Viruses

### 2.1. The Four Cardinal Hallmarks of ICD

**Figure 1:**
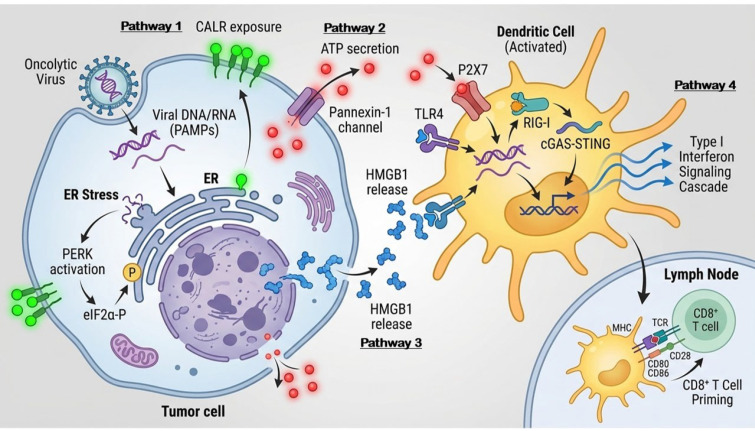
Molecular Mechanisms of Immunogenic Cell Death (ICD) Induced by Oncolytic Viruses.

Immunogenic cell death represents a specialized form of regulated cell death that stimulates adaptive immune responses against dead-cell antigens, fundamentally distinguishing it from apoptosis, necrosis, or other “immunologically silent” modes of cell demise [30,31]. The induction of ICD by oncolytic viruses relies on four cardinal hallmarks that occur in precise spatiotemporal coordination (Figure 1): (1) pre-apoptotic surface exposure of calreticulin, (2) secretion of ATP, (3) passive release of HMGB1, and (4) production of type I interferons [30,31,32,33,34,35,36,37,38].

Molecular mechanisms of immunogenic cell death (ICD) induced by oncolytic viruses. Oncolytic virus infection of cancer cells triggers spatiotemporally coordinated release of four cardinal damage-associated molecular patterns (DAMPs): (Pathway 1) Calreticulin (CRT) exposure on cell surfaces (6–12 h) serving as “eat me” signal binding CD91 on dendritic cells (DCs); (Pathway 2) ATP secretion (12–24 h) through pannexin-1 channels binding P2X7/P2Y2 receptors creating DC chemotactic gradients and triggering inflammasome activation; (Pathway 3) HMGB1 release (24–48 h) upon membrane permeabilization engaging TLR4/RAGE receptors; (Pathway 4) Type I interferon (IFN-I) production (6–24 h) through RIG-I/MDA5 and cGAS-STING pathway activation. Unique to viral ICD is simultaneous release of viral pathogen-associated molecular patterns (PAMPs) including dsRNA, 5′-triphosphate RNA, and viral proteins recognized by TLR3/7/8 and cytoplasmic sensors. This synergistic DAMP + PAMP release creates extraordinarily immunostimulatory milieu driving robust DC recruitment, maturation, tumor antigen cross-presentation, and CD8+ T cell priming, culminating in systemic anti-tumor immunity that transforms immunologically “cold” tumors into “hot” immune-inflamed microenvironments.

HALLMARK 1: Calreticulin Exposure (6–12 h post-infection)

Calreticulin (CRT), an endoplasmic reticulum (ER) chaperone protein, translocates to the cell surface early during ICD, serving as an “eat me” signal for dendritic cells [35,36]. This ER stress response is triggered by viral replication-associated protein misfolding, nutrient depletion, and activation of the unfolded protein response [39,40,41,42]. Surface CRT binds CD91 on dendritic cells, initiating phagocytosis of dying tumor cells and subsequent antigen cross-presentation [43,44]. The molecular machinery involves ER-to-Golgi transport of CRT associated with ERp57, followed by SNARE-dependent fusion of CRT-containing vesicles with the plasma membrane [40,41,45,46]. Critically, CRT exposure must occur pre-apoptotically—externalized phosphatidylserine (a late apoptotic marker) [47,48,49,50,51] delivers “don’t eat me” signals that inhibit phagocytosis, necessitating CRT exposure before caspase-3 activation [35,36,42].

HALLMARK 2: ATP Secretion (12–24 h post-infection)

Extracellular ATP acts as a “find me” signal, recruiting dendritic cell precursors and monocytes to sites of ICD through purinergic P2Y2 and P2X7 receptor activation [52,53,54,55]. Virus-infected cells secrete ATP through pannexin-1 channels, which open in response to caspase-3/7 activation and viral inhibition of cellular energy metabolism [55,56]. Released ATP triggers multiple pro-inflammatory cascades: P2X7 activation on dendritic cells induces NLRP3 inflammasome assembly, leading to IL-1β maturation and secretion [52,53]. IL-1β, in turn, stimulates chemokine production (CCL2, CXCL1) that amplifies immune cell recruitment [57]. The ATP concentration gradient (millimolar intracellularly versus nanomolar extracellularly) ensures robust “find me” signaling even from small numbers of dying cells [58,59].

HALLMARK 3: HMGB1 Release (24–48 h post-infection)

High-mobility group box 1 (HMGB1), a nuclear DNA-binding protein, is passively released from necrotic or late apoptotic tumor cells during oncolytic virus infection [60,61]. Extracellular HMGB1 functions as an archetypal DAMP, binding Toll-like receptor 4 (TLR4) and receptor for advanced glycation end products (RAGE) on dendritic cells [60,62,63,64,65]. TLR4 engagement by HMGB1 is essential for dendritic cell maturation and efficient cross-priming of CD8+ T cells against tumor antigens [60,66]. The redox state of HMGB1 determines its immunological function: reduced HMGB1 acts as a chemoattractant, oxidized HMGB1 exhibits anti-inflammatory properties, while disulfide HMGB1 optimally activates TLR4 signaling [64]. Tumors resistant to anthracycline chemotherapy often exhibit defects in HMGB1 release or downstream TLR4 signaling, emphasizing its critical role in therapeutic immunity [66,67].

HALLMARK 4: Type I Interferon Production (6–24 h post-infection)

Type I interferons (IFN-α/β) constitute the fourth and arguably most critical hallmark of virus-induced ICD, distinguishing oncolytic virotherapy from chemotherapy or radiation-induced ICD [68,69,70,71,72,73]. For RNA viruses, viral replication intermediates—including double-stranded RNA and 5′-triphosphate RNA—are detected by the cytoplasmic RNA sensors retinoic acid-inducible gene I (RIG-I) and melanoma differentiation-associated gene 5 (MDA5), which signal through the mitochondrial antiviral signaling protein (MAVS) adaptor to activate IRF3/IRF7 and induce robust type I interferon production [32,33,34,68]. For DNA viruses, cytoplasmic viral DNA is sensed by cyclic GMP-AMP synthase (cGAS), which produces the second messenger cyclic GMP-AMP (cGAMP) to activate stimulator of interferon genes (STING) and TANK-binding kinase 1 (TBK1), ultimately leading to IRF3/IRF7-dependent IFN-I production [32,33,34,68]. Importantly, these pattern recognition receptor pathways induce not only type I interferons (IFN-α/β) but also type III interferons (IFN-λ family), which share overlapping signaling pathways through the JAK-STAT cascade and exert complementary antiviral and immunomodulatory functions [74]. Furthermore, IFN-I and IFN-III can stimulate the production of type II interferon (IFN-γ) following innate immune activation, although direct evidence of this remains limited [75]. This layered interferon response—from innate IFN-I/III to adaptive IFN-γ production—coordinates antiviral immunity by bridging innate and adaptive immune mechanisms, and may be particularly relevant to the sustained antitumor immune responses observed following oncolytic virus-mediated immunogenic cell death. IFN-I serves multiple immunostimulatory functions: (1) direct activation of dendritic cells, enhancing their capacity for antigen cross-presentation; (2) promotion of T cell priming and survival; (3) upregulation of MHC-I expression on tumor cells; and (4) activation of natural killer cells [69,70,71,72,73,76,77,78]. Critically, CD8α+ dendritic cells require IFN-I signaling to efficiently cross-present tumor antigens to CD8+ T cells—mice deficient in the IFN-I receptor fail to mount anti-tumor immunity following immunogenic chemotherapy or oncolytic virus therapy [69,70].

### 2.2. Spatiotemporal Coordination and Synergy Between PAMPs and DAMPs

The defining feature of virus-induced ICD is the spatiotemporally orchestrated release of DAMPs alongside viral PAMPs, creating a synergistic “danger signal” that far exceeds the immunogenicity of either component alone [30,31]. This PAMP + DAMP synergy resolves the fundamental challenge facing the innate immune system: distinguishing self from non-self in the absence of microbial patterns [79,80].

Calreticulin exposure at 6–12 h initiates dendritic cell recruitment when viral replication is actively generating PAMPs (viral RNA, DNA). ATP secretion at 12–24 h sustains dendritic cell influx precisely when viral titers peak and tumor antigen availability is maximal. HMGB1 release at 24–48 h provides the final TLR4-dependent maturation signal for dendritic cells already laden with tumor antigens [60,66]. Type I interferons produced throughout infection (6–24 h) ensure dendritic cells are optimally conditioned for cross-presentation [69,70,71,72,73].

This temporal coordination is not coincidental—viral replication kinetics naturally align DAMP exposure with peak PAMP production, creating an “immunological window” during which dendritic cells encounter the complete constellation of danger signals necessary for robust T cell priming [30,81]. Chemotherapy-induced ICD lacks viral PAMPs and generates 10–100-fold lower IFN-I levels, presumably explaining its inferior immunogenicity compared to oncolytic viruses [68,73,81].

## 3. Oncolytic Virus Platforms: Comparative Analysis

### 3.1. DNA Virus Platform Overview

**Figure 2:**
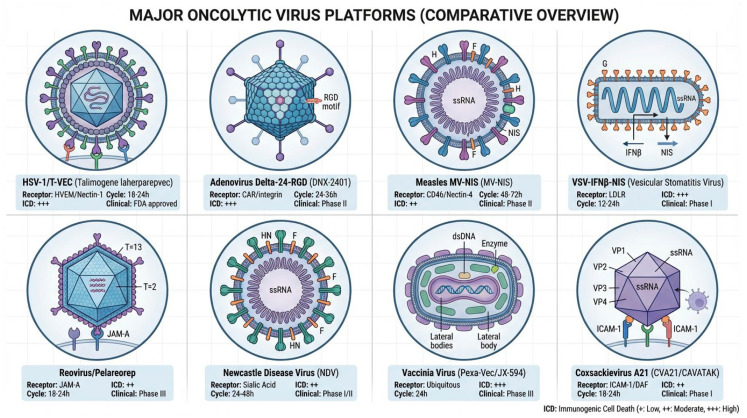
Comparative Oncolytic Virus Platforms and Clinical Synergy with Immune Checkpoint Inhibitors.

The major oncolytic virus platforms are summarized in Figure 2, with their comparative ICD characteristics detailed in Table 1. DNA virus platforms, including Vaccinia virus (Poxviridae), Herpes Simplex Virus-1 (Herpesviridae), and Adenovirus (Adenoviridae), have been extensively developed as oncolytic therapeutics due to their large genome size and capacity to accommodate substantial transgenes. These platforms offer substantial transgene capacity: oncolytic adenoviral vectors retaining replication-essential genes accommodate approximately 7–8 kb of transgene inserts, while third-generation helper-dependent (“gutless”) adenoviral vectors, in which all viral coding sequences are removed, can accommodate up to approximately 35 kb of foreign DNA. The wild-type Vaccinia virus genome spans approximately 190 kb; however, clinical vector systems employ modified genomes incorporating specific gene deletions (e.g., thymidine kinase, vaccinia growth factor, B18R) that reduce the genome size while creating space for multiple immunostimulatory transgenes, with an effective transgene capacity exceeding 25 kb [82,83,84,85]. However, a common limitation across DNA virus platforms is the presence of viral genes encoding interferon antagonist proteins that evolved to evade host antiviral responses. Vaccinia virus encodes B18R (a soluble type I interferon receptor decoy), E3L (a dsRNA-binding protein that blocks RIG-I and MDA5 signaling), and K3L (a PKR inhibitor), which collectively suppress interferon production and signaling in infected cells [86,87,88,89]. Similarly, HSV-1 expresses ICP34.5 and ICP47, while Adenovirus produces E1A and E3 proteins, all of which contribute to interferon evasion [90,91,92,93,94]. These viral countermeasures result in 10–100-fold lower type I interferon production compared to RNA viruses, potentially reducing the immunogenicity of DNA virus-induced cell death despite their capacity for direct oncolysis [86,87].Comparative oncolytic virus platforms and clinical synergy with immune checkpoint inhibitors. DNA virus platforms include Vaccinia virus (large 190 kb genome enabling extensive transgene insertion, clinical development in HCC), HSV-1 (T-VEC, first FDA-approved oncolytic virus in 2015 for melanoma with 16.3% durable response rate), and adenovirus (first approved in China 2005, CG0070 achieving 47% complete response in bladder cancer). RNA virus platforms feature Coxsackieviruses with smaller genomes (7.4 kb) but faster replication (12–24 h vs. 24–48 h for DNA viruses) and superior type I interferon production (10-fold higher). CVA21 (CAVATAK/V937) utilizes ICAM-1 receptor achieving 28% confirmed objective response rate (ORR) as monotherapy and 47% ORR when combined with pembrolizumab (CAPRA trial). CVB3 with innovative microRNA-targeting (miR-1/miR-133) achieves cardiac attenuation while preserving complete tumor oncolytic efficacy, representing paradigm-shifting safety engineering. Additional emerging platforms, including Newcastle disease virus (NDV), are discussed in Section 4.4. The symbols “++” and “+++” indicate moderate and high capability of ICD induction, respectively.

### 3.2. Vaccinia Virus (Poxviridae)

Vaccinia virus, a large double-stranded DNA poxvirus with a 190 kb genome, represents one of the most extensively engineered oncolytic platforms due to its exceptional transgene capacity (greater than 25 kb), well-characterized replication biology, and cytoplasmic replication cycle that avoids nuclear host defense mechanisms [83,84,85,95]. The thymidine kinase gene deletion strategy emerged as the first rational attenuation approach, exploiting the differential nucleotide metabolism between quiescent normal cells and rapidly dividing tumor cells. Thymidine kinase-deleted Vaccinia virus cannot replicate efficiently in normal cells with low endogenous nucleotide pools but replicates robustly in tumor cells with dysregulated nucleotide metabolism driven by oncogenic transformation [82,96,97,98].

JX-594, also known as Pexastimogene devacirepvec or Pexa-Vec, represents the lead clinical candidate in the Vaccinia virus platform. This construct incorporates TK deletion for tumor selectivity along with expression cassettes for human granulocyte-macrophage colony-stimulating factor (GM-CSF) and the *lacZ* reporter gene [99,100,101,102]. Early phase I trials demonstrated dose-dependent viral replication, transgene expression, and anti-tumor efficacy with manageable safety profiles [99,100]. The phase II TRAVERSE trial randomized 129 patients with advanced hepatocellular carcinoma to receive either high-dose or low-dose intravenous JX-594, demonstrating significant dose-dependent survival benefit with median overall survival of 14.1 months in the high-dose arm versus 6.7 months in the low-dose arm [101,102]. Based on these encouraging results, a phase III trial (PHOCUS) was initiated comparing JX-594 plus sorafenib versus sorafenib alone in hepatocellular carcinoma patients. However, this pivotal trial was terminated early for futility when interim analysis revealed no survival benefit from the addition of JX-594 to standard sorafenib therapy [101].

The clinical failure of the PHOCUS trial highlighted several critical challenges facing the Vaccinia virus platform. First, the natural hepatotropism of poxviruses results in dose-limiting liver toxicity, restricting the achievable viral doses. Second, widespread vaccination programs during the smallpox eradication era left 30–50% of older populations with pre-existing anti-vaccinia neutralizing antibodies that rapidly clear systemically administered virus, dramatically reducing tumor biodistribution [101,102,103,104]. Third, despite GM-CSF expression intended to enhance dendritic cell recruitment and activation, the extensive interferon evasion mechanisms encoded by Vaccinia virus (B18R, E3L, K3L) substantially blunt the type I interferon response critical for robust dendritic cell activation and CD8+ T cell priming. Comparative studies have demonstrated that Vaccinia-infected tumor cells generate 10–50-fold lower IFN-β levels compared to RNA virus-infected cells, correlating with reduced tumor-infiltrating lymphocyte recruitment and inferior anti-tumor immunity in preclinical models [68,105,106,107,108,109,110,111].

Despite these setbacks, next-generation Vaccinia platforms continue to be developed with improved designs. Deletion of the B18R interferon antagonist gene enhanced anti-tumor immunity in preclinical models, although this modification also reduced viral replication efficiency, necessitating a careful balance between safety, replication, and immunogenicity [86]. Engineering strategies incorporating immunostimulatory transgenes such as interleukin-12, interleukin-7, or bispecific T cell engager molecules have shown promise in enhancing tumor regression in murine models [112,113,114]. The double-deleted Vaccinia construct VV-DD (lacking both TK and vaccinia growth factor [VGF] genes—VGF is a secreted epidermal growth factor homolog that promotes paracrine mitogenic signaling in neighboring cells, and its deletion restricts viral replication to tumor cells with constitutively activated epidermal growth factor receptor/RAS pathways) achieved safe systemic administration in phase I trials with evidence of tumor-specific viral replication, although clinical efficacy data remain limited [115,116].

### 3.3. Herpes Simplex Virus-1 (Herpesviridae)

Herpes Simplex Virus-1 (HSV-1), a 150 kb double-stranded DNA alphaherpesvirus, has emerged as the most clinically successful oncolytic virus platform to date, culminating in regulatory approval of talimogene laherparepvec (T-VEC) by the United States Food and Drug Administration (FDA) in 2015 and by the European Medicines Agency in the same year [20,90,91]. HSV-1 naturally infects epithelial cells and establishes latency in sensory neurons, providing inherent neurotropism that necessitates careful genetic attenuation for therapeutic applications [91]. T-VEC incorporates three critical genetic modifications designed to enhance tumor selectivity while maintaining oncolytic potency and adding immunostimulatory functions. First, deletion of both copies of the ICP34.5 gene (also known as γ34.5) restricts viral replication to tumor cells with defective protein kinase R (PKR) pathways, as ICP34.5 normally counteracts PKR-mediated translational shutdown during viral infection [20,91]. Tumor cells frequently harbor mutations in the PKR pathway due to oncogenic transformation, allowing selective T-VEC replication. Second, deletion of the ICP47 gene enhances major histocompatibility complex class I antigen presentation by preventing ICP47-mediated inhibition of the transporter associated with antigen processing (TAP), thereby improving tumor antigen presentation to CD8+ T cells. Third, insertion of the human GM-CSF gene under control of a strong promoter drives local cytokine production at the tumor site, promoting dendritic cell recruitment, maturation, and subsequent T cell priming [20,91,92].

The pivotal OPTiM phase III trial randomized 436 patients with unresectable stage IIIB-IV melanoma to receive either intralesional T-VEC or subcutaneous GM-CSF control [20,117]. The trial met its primary endpoint, demonstrating a significantly improved durable response rate (defined as an objective response lasting continuously for at least 6 months) of 16.3% in the T-VEC arm compared to 2.1% in the GM-CSF arm (*p* < 0.001). Median overall survival showed a trend toward improvement at 23.3 months versus 18.9 months, although this difference did not achieve statistical significance in the primary analysis [20,117]. Notably, 64% of durable responders exhibited abscopal responses (i.e., tumor regression at distant, non-injected sites mediated by systemic anti-tumor immunity) in non-injected lesions, indicating the development of systemic anti-tumor immunity capable of controlling distant metastases [118]. Subgroup analyses revealed that patients with earlier-stage disease (stage IIIB/C and stage IVM1a) derived greater benefit from T-VEC compared to those with visceral metastases [118].

The true therapeutic potential of T-VEC has been realized through rational combination with immune checkpoint inhibitors. A phase II trial combining T-VEC with ipilimumab (anti-CTLA-4) in previously untreated melanoma patients demonstrated an objective response rate of 39% compared to historical ipilimumab monotherapy response rates of approximately 18%, representing more than doubling of efficacy [119]. The MASTERKEY-265 phase Ib/III trial (ClinicalTrials.gov: NCT02263508) evaluated T-VEC in combination with pembrolizumab (anti-PD-1) in melanoma patients. The phase Ib portion (N = 21) achieved a remarkable objective response rate of 62% with a complete response rate of 33% and three-year overall survival of 71%. However, the subsequent randomized phase III portion (N = 692) failed to meet its co-primary endpoints of progression-free survival and overall survival, with an objective response rate (ORR) of 48.6% versus 41.3% for pembrolizumab alone (CR 17.9% vs. 11.6%) [26]. Mechanistic studies from these combination trials revealed that T-VEC increases CD8+ T cell infiltration into tumors, induces inflammatory gene signatures, and upregulates PD-L1 expression on tumor cells and immune cells within the tumor microenvironment. Importantly, this T-VEC-induced PD-L1 upregulation represents an adaptive immune resistance mechanism: the influx of activated T cells producing interferon-γ drives PD-L1 expression as a physiological negative feedback response, which paradoxically limits the anti-tumor efficacy of T-VEC monotherapy. This observation provides the mechanistic rationale for combining T-VEC with PD-1/PD-L1 checkpoint inhibitors—by blocking the PD-1/PD-L1 axis that is upregulated in response to virus-induced inflammation, the combination therapy unleashes the full cytotoxic potential of the newly recruited tumor-infiltrating T cells [120]. Serial tumor biopsies demonstrated that T-VEC converts immune-excluded “cold” tumors—defined as tumors with minimal immune cell infiltration, low inflammatory gene expression, and an immunosuppressive microenvironment—into inflamed “hot” tumors, characterized by high densities of tumor-infiltrating lymphocytes, active inflammatory signaling, and a microenvironment permissive for anti-tumor immune responses. Specifically, post-treatment biopsies revealed dense CD8+ T cell infiltration, activated dendritic cells, and upregulation of interferon-γ-inducible genes [26,120].

Beyond T-VEC, additional HSV-1 platforms have achieved clinical success in specific indications. G47Δ, a triple-deleted HSV-1 construct lacking ICP34.5, ICP6, and α47 genes, received conditional approval in Japan in 2021 for treatment of recurrent glioblastoma based on a phase II trial demonstrating an 84% one-year survival rate, a substantial improvement over historical controls showing approximately 15% one-year survival [121]. The unique triple-deletion strategy employed in G47Δ enhances both safety and immunogenicity while maintaining robust oncolytic activity against glioblastoma cells [121]. Earlier-generation constructs such as G207 have also shown promising safety and preliminary efficacy signals in combination with radiation therapy for recurrent glioblastoma [122].

Despite these clinical successes, HSV-1 platforms face important limitations. The high seroprevalence of HSV-1 antibodies in the general population (60–90% depending on geographic region and age) may reduce therapeutic efficacy through antibody-mediated viral neutralization, although the impact of pre-existing immunity on clinical outcomes remains incompletely characterized. The ICP34.5 deletion, while critical for safety, also reduces the virus’s capacity to induce robust type I interferon responses, potentially limiting immunogenicity compared to RNA virus platforms that lack interferon antagonist genes [90,91].

### 3.4. Adenovirus (Adenoviridae)

Adenoviruses are non-enveloped, double-stranded DNA viruses with relatively compact 36 kb genomes that offer advantages of high infectivity, efficient gene transfer capability, and well-established manufacturing processes that facilitate clinical-scale production [93,94]. The development of oncolytic adenoviruses has focused primarily on engineering tumor-selective replication through deletion or modification of viral genes required for replication in normal but not transformed cells. The E1B-55K gene deletion strategy emerged as the first tumor-selective approach, based on the rationale that E1B-55K normally inactivates the p53 tumor suppressor protein, and therefore E1B-deleted adenoviruses should replicate selectively in p53-deficient tumor cells which comprise approximately 50% of human cancers [123,124]. However, subsequent mechanistic studies revealed that the selectivity of E1B-deleted adenoviruses does not strictly depend on p53 status, and the precise mechanisms underlying their tumor selectivity remain incompletely understood [123,124].

H101, an E1B-55K-deleted adenovirus, achieved regulatory approval in China in 2005 for treatment of head and neck cancer when administered intratumorally in combination with chemotherapy, representing the first oncolytic virus to receive regulatory approval in the world [123,124]. Clinical trials in Chinese patients demonstrated that intratumoral injection of H101 combined with chemotherapy produced superior response rates compared to chemotherapy alone in patients with advanced head and neck squamous cell carcinoma [123]. However, H101 has not been approved outside of China, in part due to questions regarding the mechanistic basis for its tumor selectivity and limited availability of randomized controlled trial data meeting Western regulatory standards [124].

CG0070 represents a more sophisticated adenovirus platform incorporating both tumor-selective replication elements and immunostimulatory transgene expression. This construct places the essential E1A gene under control of the E2F-1 promoter, restricting viral replication to cells with retinoblastoma (Rb) pathway defects (present in approximately 70% of cancers), and expresses human GM-CSF to enhance dendritic cell recruitment and maturation [125,126]. In a phase I trial enrolling patients with BCG-unresponsive non-muscle-invasive bladder cancer—a population with extremely limited therapeutic options—CG0070 demonstrated a complete response rate of 47%, representing the highest efficacy reported for this challenging indication [125,126]. The durability of responses and favorable safety profile have supported advancement into phase II/III development for bladder cancer.

Additional adenovirus platforms under clinical investigation include DNX-2440, an OX40 ligand-expressing conditionally replicating adenovirus showing promising safety and preliminary efficacy signals in phase I trials for recurrent glioblastoma [127], and VCN-01, a hyaluronidase-expressing adenovirus designed to degrade the hyaluronic acid-rich tumor stroma characteristic of pancreatic adenocarcinoma, thereby enhancing viral spread and T cell infiltration [128]. Preclinical studies of VCN-01 demonstrated improved viral dissemination throughout desmoplastic pancreatic tumors and enhanced survival in orthotopic murine models, with phase I clinical trials ongoing [128,129].

Despite these advances, adenovirus platforms face several inherent limitations. The natural hepatotropism of adenovirus serotype 5 (the most commonly used serotype for oncolytic applications) causes dose-limiting liver toxicity when administered systemically, restricting clinical applications primarily to locoregional delivery approaches [93,94]. High seroprevalence of neutralizing antibodies against adenovirus serotype 5 in human populations (50–90% in most geographic regions) substantially reduces the efficacy of systemically administered virus through rapid antibody-mediated clearance [93,94]. The tight junction localization of the coxsackievirus–adenovirus receptor (CAR) in normal epithelia limits viral spread through intact tissues, although this barrier is often disrupted in tumor microenvironments [130,131]. Alternative adenovirus serotypes with different receptor tropisms and lower seroprevalence are under investigation to address these limitations [93,94].

## 4. RNA Virus Platforms

### 4.1. Coxsackievirus A21 (CVA21)

Coxsackievirus A21 (CVA21), a naturally occurring picornavirus with a compact 7.4 kb positive-sense single-stranded RNA genome, has emerged as one of the most promising oncolytic virus platforms based on exceptional preclinical immunogenicity and remarkable clinical efficacy when combined with checkpoint inhibitors [132,133,134,135,136,137]. CVA21 belongs to the Enterovirus genus within the Picornaviridae family and exhibits tumor selectivity through its unique dependence on intercellular adhesion molecule-1 (ICAM-1, also known as CD54) for cellular entry [137,138,139]. Unlike most enteroviruses that utilize coxsackievirus–adenovirus receptor (CAR) as their primary receptor, CVA21 binds ICAM-1 with high affinity and requires ICAM-1 for productive infection and viral replication [135].

ICAM-1 is a cell surface glycoprotein normally expressed at low levels on epithelial cells but is dramatically upregulated on multiple cancer types in response to inflammatory signaling (mediated by TNF-α, IL-1β, and IFN-γ), hypoxic stress (through HIF-1α transcriptional activation), oncogenic mutations (particularly in RAS and BRAF pathways), and loss of tumor suppressor function [137,138,139]. Melanoma, bladder cancer, prostate cancer, breast cancer, and multiple myeloma commonly exhibit 10–100-fold overexpression of ICAM-1 compared to corresponding normal tissues, providing CVA21 with inherent tumor tropism without requiring genetic engineering [137,138,139]. The molecular mechanism of CVA21 entry involves a multi-step process: initial ICAM-1 binding triggers conformational changes in the viral capsid that expose hydrophobic regions of the VP1 capsid protein, leading to membrane penetration and delivery of the viral RNA genome into the cytoplasm within 30–60 min, substantially faster than the 2–8 h entry kinetics characteristic of DNA viruses [135].

The exceptional immunogenicity of CVA21 stems from its minimal interferon evasion capacity. Unlike DNA viruses that encode multiple interferon antagonist proteins, picornaviruses including CVA21 lack genes for interferon antagonists, resulting in robust type I interferon production in infected cells, representing more than 10-fold higher levels than those generated by Vaccinia, HSV-1, or Adenovirus infections [132,133,136]. This profound IFN-I response drives extensive dendritic cell activation, upregulation of major histocompatibility complex class I molecules on tumor cells, production of T cell-recruiting chemokines (CXCL9, CXCL10), and activation of natural killer cells [132,133].

The CALM phase II trial established CVA21’s clinical activity as monotherapy in melanoma. Fifty-seven patients with unresectable stage IIIC-IVM1c melanoma received intratumoral CVA21 (marketed under the brand name CAVATAK) administered on days 1, 3, 5, 8, and 22 of each 28-day cycle [140]. The trial demonstrated a confirmed objective response rate of 28.1% (unconfirmed ORR 38.6% by irRECIST) with a durable response rate of 21.1%, and median overall survival of 26 months in this heavily pre-treated population. Importantly, 26.7% of objective responses occurred in target non-injected distant lesions, demonstrating the development of systemic anti-tumor immunity and abscopal effects [140].

The CAPRA trial represents a key achievement in oncolytic virotherapy. This phase Ib study (NCT02565992) enrolled 36 patients with advanced melanoma (stage IIIB-IV) to receive intratumoral V937 (CVA21) combined with intravenous pembrolizumab (2 mg/kg every 3 weeks) [141]. The combination achieved an objective response rate of 47% (17 of 36 patients), including a complete response rate of 22% (8 patients) and a partial response rate of 25% (9 patients), with 82% of responders maintaining responses for 6 months or longer [141]. These results compared favorably to historical benchmarks for pembrolizumab monotherapy (Table 2), and responses were observed even in patients previously treated with immunotherapy (3 of 8 responded). The toxicity profile of CVA21 plus pembrolizumab was generally manageable. Grade 3–5 treatment-related adverse events occurred in 14% of patients (5 of 36), with no grade 5 (fatal) events [141]. The safety profile was comparable to pembrolizumab monotherapy, suggesting that CVA21 does not substantially increase the risk of severe immune-related toxicities when combined with PD-1 blockade.

The separate phase 1b MITCI study (NCT02307149) evaluated intratumoral V937 combined with ipilimumab in 50 patients, achieving an ORR of 30% overall and 47% in anti-PD-1-naive patients. Notably, tumor regression occurred in both injected and non-injected lesions. Median immune-related PFS was 6.2 months and median OS was 45.1 months. Common treatment-related AEs included pruritus (50%), fatigue (44%), and diarrhea (32%). No V937-related dose-limiting toxicities or grade 5 AEs occurred; grade 3–4 AEs (14%, all ipilimumab-related) included dehydration, diarrhea, and hepatotoxicity (4% each) [142].

Beyond melanoma, CVA21 has demonstrated clinical activity in bladder cancer. The phase I CANON trial evaluated intravesical CVA21 administration in 15 patients with BCG-unresponsive non-muscle-invasive bladder cancer [143]. CVA21 induced tumor inflammation, hemorrhage, and complete tumor resolution in 1 patient after single or multiple intravesical doses, without significant toxicity. It produced marked inflammatory changes in NMIBC biopsies, upregulating IFN-inducible genes such as PD-L1, LAG3, Th1-associated chemokines, and the innate activator RIG-I compared with untreated tumors. [143]. These results support expansion into phase II development for bladder cancer, particularly in combination with checkpoint inhibitors.

**Table 2:** Clinical Trials of Oncolytic Viruses Combined with Immune Checkpoint Inhibitors and Tyrosine Kinase Inhibitor (TKI).

Trial	Oncolytic Virus	Checkpoint Inhibitor	Cancer Type	Phase	N	ORR (%)	CR (%)	Key Findings	Year	Ref.
OPTiM	T-VEC (Herpesvirus: HSV-1)	Mono (vs. GM-CSF)	Stage IIIB-IV melanoma	III	436	26	11	16.3% DRR; FDA approval basis	2015	[20,117]
—	T-VEC (Herpesvirus: HSV-1)	Ipilimumab	Unresectable melanoma	II	198	39	13	2× vs. ipi alone (18%); abscopal effects	2016	[25,119]
MASTERKEY-265	T-VEC (Herpesvirus: HSV-1)	Pembrolizumab	Treatment-naive melanoma	Ib/III	21 (Ib); 692 (III)	62 (Ib); 48.6 (III)	33 (Ib); 17.9 (III)	71% 3-yr OS (Ib); Phase III failed primary PFS/OS endpoints	2021	[26,144]
G47Δ (Delytact)	G47Δ/Teserpaturev (Herpesvirus: HSV-1)	Monotherapy	Malignant glioma	II	19	—	—	84% 1-yr OS (vs. 15% hist.); Japan approval 2021	2022	[121]
—	CG0070 (Adenovirus: HAdV-5)	Pembrolizumab	BCG-unresp. NMIBC	II	43	47	47	High CR in bladder cancer	2018	[125,126]
—	DNX-2440 (Adenovirus: HAdV-5)	Pembrolizumab	Recurrent GBM	I/II	Ongoing	—	—	OX40L expression; local immune activation	2023	[127]
—	VCN-01 (Adenovirus: HAdV-5)	Pembrolizumab	Pancreatic adeno.	I	12	33	—	Hyaluronidase stroma degradation	2022	[128,129]
PHOCUS	Pexa-Vec/JX-594 (Poxvirus: Vaccinia virus)	Sorafenib	Advanced HCC	III	129	—	—	Terminated for futility	2019	[102]
CALM	CVA21/CAVATAK (Enterovirus: Coxsackievirus A21)	Monotherapy	Stage IIIC/IV melanoma	II	57	28.1	—	mOS 26 mo; injected + non-injected responses; ICD biomarkers	2019	[140]
CAPRA	CVA21/V937 (Enterovirus: Coxsackievirus A21)	Pembrolizumab	Advanced melanoma	Ib	36	47	22	Increased serum CXCL10 and CCL22,	2020	[141]
Duke Phase I (NCT01491893)	PVSRIPO (Picornavirus: Poliovirus–Rhinovirus chimera)	Monotherapy	Recurrent GBM	I	31	—	—	21% 36-mo survival (vs. 4% hist.); BT designation	2022	[145]

Legend: ORR = objective response rate; CR = complete response; OS = overall survival; HCC = hepatocellular carcinoma; NMIBC = non-muscle invasive bladder cancer; BCG = Bacillus Calmette-Guérin.

### 4.2. Coxsackievirus A11 (CVA11)

Coxsackievirus A11 (CVA11) is gaining attention as a next-generation oncolytic enterovirus with activity against multiple solid tumor types. Although clinical translation has not yet been achieved, three independent preclinical studies in lung cancer, malignant pleural mesothelioma, and colorectal cancer provide converging evidence that CVA11 exerts both direct cytolytic and immunomodulatory antitumor effects [146,147,148].

In a comprehensive preclinical investigation, CVA11 was evaluated in several human NSCLC cell lines and xenograft mouse models [146]. CVA11 infection caused extensive oncolytic activity in multiple human NSCLC cell lines, with high intercellular adhesion molecule-1 (ICAM-1) expression associated with greater CVA11-induced cytotoxicity. In vitro inhibition analysis using a pan-caspase inhibitor and Western blot detection of cleaved poly (ADP-ribose) polymerase (PARP) indicated that apoptosis partly contributed to CVA11-driven cytotoxicity. CVA11 infection-induced immunogenic cell death in vitro was strongly suggested by substantial calreticulin expression and release of high mobility group box-1 protein (HMGB1). In vivo, repeated intratumoral administration led to significant suppression of tumor growth compared with control cohorts, and complete tumor regression was observed in a subset of animals. Importantly, systemic toxicity was minimal, with no significant body-weight loss or histologic injury in major organs [146].

A separate study from the same group examined CVA11 in human malignant pleural mesothelioma (MPM) models and provided mechanistic insights into viral tropism [148]. The study identified intercellular adhesion molecule-1 (ICAM-1) as a key determinant of viral entry. Tumor cells expressing high ICAM-1 levels were markedly more susceptible to infection and lysis, whereas ICAM-1–low cells showed limited viral replication. Blocking antibodies against ICAM-1 reduced viral infectivity, confirming receptor dependence [148]. CVA11 infection activated ERK and Akt signaling pathways in permissive cells, which appeared to facilitate efficient viral replication [148]. In SCID mouse xenograft models, intratumoral administration resulted in significant tumor growth inhibition without overt toxicity [148]. Notably, treated tumors exhibited both immunostimulatory changes (increased inflammatory mediators, enhanced immune cell infiltration) and counter-regulatory adaptive responses, including upregulation of PD-L1. This dual effect reflects the complex interplay between immune activation and adaptive immune resistance within the tumor microenvironment: while CVA11 infection promotes a pro-inflammatory milieu through lytic replication and innate immune activation, the resulting interferon signaling simultaneously upregulates PD-L1 as a physiological feedback mechanism to restrain excessive immune responses. Importantly, this virus-induced PD-L1 upregulation, rather than being detrimental, creates a therapeutic vulnerability that can be exploited by subsequent immune checkpoint blockade. The authors proposed that viral infection converts poorly inflamed tumors toward a more immunologically active phenotype, and the concurrent PD-L1 upregulation provides a strong rationale for combination strategies with anti-PD-1/PD-L1 antibodies.

An earlier report explored CVA11 in colorectal cancer, focusing on oxaliplatin-resistant colorectal cancer (CRC) models [147]. The investigators hypothesized that chemotherapy could alter antiviral defense pathways and thereby increase tumor susceptibility to oncolytic infection. Indeed, pretreatment with oxaliplatin enhanced viral cytopathic effects in resistant CRC cell lines [147]. In xenograft experiments, sequential administration of oxaliplatin followed by CVA11 produced greater tumor suppression than either therapy alone [147]. Mechanistically, chemotherapy appeared to attenuate intrinsic antiviral responses within tumor cells, enabling more robust viral replication [147]. This study introduced a sequence-dependent combinatorial strategy in which standard cytotoxic therapy primes tumors for enhanced oncolytic efficacy.

Across these tumor types, several shared principles emerge. First, CVA11 displays selective replication in malignant cells with limited off-target toxicity in animal models [146,147,148]. Second, receptor expression—particularly ICAM-1—contributes to tumor tropism and may serve as a predictive biomarker [148]. Third, CVA11 infection consistently induces inflammatory and immune-related gene expression changes, suggesting immunogenic consequences beyond direct lysis [148]. Finally, therapeutic synergy with other modalities is plausible, including chemotherapy-induced sensitization [147] and checkpoint inhibitor combination strategies [146,148].

However, all available data derive from immunodeficient xenograft systems, limiting assessment of adaptive antitumor immunity. Host antiviral immunity, neutralizing antibodies, and systemic delivery challenges remain unresolved. No peer-reviewed clinical trials have yet evaluated CVA11 in cancer patients.

### 4.3. Coxsackievirus B3 (CVB3): Genetic Innovation in microRNA-Targeted Safety Engineering

Miyamoto et al. demonstrated that wild-type Coxsackievirus B3 (CVB3), closely related to CVA21 within the Enterovirus genus, functions as a potent oncolytic virus with robust immunostimulatory properties against lung adenocarcinoma [149]. This foundational work demonstrated that CVB3 infection induces all four cardinal hallmarks of immunogenic cell death: calreticulin exposure beginning at 6–8 h post-infection, ATP secretion peaking at 12–16 h, and HMGB1 release occurring at 18–24 h [149]. Critically, the study established that intratumoral CVB3 administration markedly recruited natural killer cells and granulocytes, both of which contributed to the antitumor effects as shown by depletion assays, macrophages, and mature dendritic cells into tumor tissues, suggesting that CVB3 is a potent and well-tolerated oncolytic agent with immunostimulatory properties active against both localized and metastatic NSCLC [149]. CVB3 utilizes coxsackievirus–adenovirus receptor (CAR) for cell entry rather than ICAM-1, providing broader tumor cell tropism since CAR is widely expressed on epithelial malignancies including lung, pancreatic, ovarian, and bladder cancers [130,150,151,152,153]. The relationship between CAR expression and malignant transformation is complex and context-dependent. While disruption of tight junctions during epithelial–mesenchymal transition can expose CAR at the cell surface in some tumor types, CAR expression is frequently downregulated or lost in advanced cancers, including bladder, ovarian, cervical, endometrial, and breast carcinomas, often correlating with tumor progression and poor prognosis [154]. This loss of CAR expression may limit CVB3 infectivity in a subset of tumors and should be considered as a potential resistance mechanism. Conversely, in certain epithelial malignancies, hypoxia-induced transcriptional activation and oncogenic signaling can upregulate CAR expression [130,153]. However, CAR is also expressed in cardiac myocytes, and wild-type CVB3 infection causes viral myocarditis, a potentially fatal complication that historically precluded clinical development of CVB3 as an oncolytic therapeutic [149,155,156,157,158,159,160]. Researchers pioneered an elegant solution to this safety liability through microRNA-targeted viral engineering, creating CVB3-miRT constructs that exploit tissue-specific microRNA expression profiles to achieve tumor selectivity while protecting vital organs [149,161,162,163,164,165,166,167,168,169]. The strategy is based on the observation that cardiac muscle and skeletal muscle express exceptionally high levels of muscle-specific microRNAs, particularly miR-1 and miR-133a, while tumor cells express these microRNAs at negligible levels [163]. By engineering the CVB3 3′-untranslated region to contain multiple tandem target sequences perfectly complementary to miR-1 and miR-133a, the viral RNA becomes subject to microRNA-mediated degradation and translational repression specifically in cardiac myocytes while remaining fully functional in tumor cells lacking these microRNAs [149,166,169].

The microRNA-targeting approach has been validated across multiple oncolytic virus platforms beyond CVB3. Edge, Falls, Brown, Lichty, Atkins, and Bell demonstrated that vesicular stomatitis virus (VSV) can be detargeted from normal brain tissue by incorporating let-7 microRNA target sequences, since let-7 is abundantly expressed in neurons but downregulated in glioblastoma cells [166,168]. Leber and colleagues applied similar strategies to oncolytic measles virus, incorporating miR-7 target sequences for brain detargeting [170]. This represents a generalizable safety engineering approach applicable to diverse oncolytic virus platforms and target organs [167]. The systematic progression from mechanistic characterization through proof-of-concept safety engineering to comprehensive in vivo validation provides a roadmap for clinical translation of microRNA-targeted oncolytic viruses.

### 4.4. Other Emerging Oncolytic Virus Platforms

In addition to the platforms discussed in detail above, several other oncolytic viruses have demonstrated significant clinical promise and are at various stages of clinical development. Recombinant poliovirus (PVSRIPO), a chimeric poliovirus–rhinovirus construct, achieved durable responses in recurrent glioblastoma patients in a landmark phase I trial, with overall survival reaching 21% at 36 months compared to 4% historical control, leading to breakthrough therapy designation by the FDA [145,171,172]. Oncolytic measles virus (MV-NIS) engineered to express the human sodium-iodide symporter enables noninvasive monitoring of viral spread through radioiodine imaging and has demonstrated clinical activity in ovarian cancer, multiple myeloma, and glioblastoma [173,174,175,176]. Oncolytic parvovirus H-1 (H-1PV) completed phase I/II trials in recurrent glioblastoma, demonstrating favorable safety, evidence of immune activation, and preliminary efficacy signals including immunogenic conversion of tumor microenvironments [177,178]. Vesicular stomatitis virus (VSV) engineered with IFN-β transgene expression has entered clinical development for solid tumors and relapsed refractory T-cell lymphoma, exploiting the rapid replication kinetics and potent immunostimulatory properties characteristic of rhabdoviruses [179,180]. Pelareorep (reovirus) has been evaluated in combination with chemotherapy across multiple tumor types including pancreatic cancer [181]. Newcastle disease virus (NDV), a negative-sense single-stranded RNA paramyxovirus, has emerged as another promising oncolytic platform owing to its natural tumor selectivity, potent type I interferon induction, and demonstrated ability to enhance anti-tumor immune responses. NDV preferentially replicates in tumor cells with defective interferon signaling pathways and has been evaluated in multiple clinical trials across melanoma, glioblastoma, and colorectal cancer, both as monotherapy and in combination with checkpoint inhibitors [182] . These diverse platforms collectively expand the therapeutic landscape and provide multiple complementary approaches toward effective oncolytic virotherapy for immunologically cold tumors [16,19,27,183].

## 5. Clinical Combinations with Immune Checkpoint Inhibitors

### 5.1. Comparative Clinical Efficacy Across Oncolytic Virus Platforms

The rational combination of oncolytic viruses with immune checkpoint inhibitors has emerged as one of the most effective therapeutic strategies in modern cancer immunotherapy, with clinical trial data demonstrating synergistic anti-tumor activity substantially exceeding that achieved with either modality as monotherapy [27]. T-VEC combined with pembrolizumab achieved an objective response rate of 62% in the phase Ib portion of the MASTERKEY-265 trial (N = 21) enrolling previously untreated melanoma patients; however, the subsequent phase III randomized trial (N = 692) did not meet its co-primary endpoints of PFS and OS (ORR 48.6% vs. 41.3% for pembrolizumab alone). This contrasts with historical pembrolizumab monotherapy response rates of approximately 33–45% in similar patient populations and T-VEC monotherapy response rates of 26% in the OPTiM trial [20,26]. The complete response rate of 33% observed with the T-VEC plus pembrolizumab combination substantially exceeds the approximately 15–20% complete response rate typically observed with pembrolizumab monotherapy in melanoma, and the three-year overall survival of 71% represents a meaningful improvement over historical benchmarks [26]. T-VEC combined with ipilimumab demonstrated an objective response rate of 39% compared to approximately 18% for ipilimumab monotherapy in treatment-naive melanoma patients, representing more than doubling of the response rate [119].

V937 (CVA21) combined with pembrolizumab in the CAPRA trial achieved a notable objective response rate of 47% with 22% complete responses in advanced melanoma patients [141]. This compares favorably to the CVA21 monotherapy response rate of 28% (confirmed ORR by irRECIST) observed in the CALM trial [140,141]. The magnitude of benefit observed with the CVA21 plus pembrolizumab combination likely reflects CVA21’s exceptionally potent type I interferon induction (approximately 10-fold higher than DNA viruses), rapid induction of PD-L1 expression on tumor cells, profound increase in CD8+ T cell infiltration, and robust generation of tumor-specific T cell responses detectable by neoantigen tetramer staining [141].

### 5.2. Molecular Mechanisms Underlying Synergy Between Oncolytic Viruses and Checkpoint Inhibitors

The synergistic anti-tumor activity observed when combining oncolytic viruses with immune checkpoint inhibitors derives from complementary and mutually reinforcing immunological mechanisms that convert immunologically “cold” tumors lacking T cell infiltration into “hot” tumors densely infiltrated by activated tumor-specific CD8+ T cells. Oncolytic viruses contribute multiple critical functions to this synergy. First, selective viral replication within tumor cells followed by cytolytic cell death releases tumor-associated antigens and neoantigens into the tumor microenvironment in a form accessible to dendritic cells for uptake, processing, and presentation to T cells [25,26,120,184]. The quantity of tumor antigens released by oncolytic virus-mediated lysis substantially exceeds that generated by physiological tumor cell turnover, providing abundant antigenic material for T cell priming. Second, immunogenic cell death induced by viral infection exposes DAMPs (calreticulin, ATP, HMGB1) and PAMPs (viral RNA, viral DNA) as described in Section 2.1, activating dendritic cells and overcoming peripheral tolerance mechanisms [22,30,31]. The spatiotemporal coordination of DAMP release with PAMP exposure (detailed in Section 2.2) creates a synergistic immunological signal triggering robust dendritic cell maturation, upregulation of costimulatory molecules (CD80, CD86), production of pro-inflammatory cytokines (IL-12, IL-1β), and efficient cross-presentation of tumor antigens to CD8+ T cells [22,30,31,81]. Third, type I interferon production induced by viral infection serves multiple immunostimulatory functions essential for anti-tumor immunity. Type I interferon directly activates dendritic cells, enhancing their capacity to cross-present antigens from dying tumor cells to naive CD8+ T cells and promoting dendritic cell migration to tumor-draining lymph nodes where T cell priming occurs [69,70,71,72,73]. Type I interferon promotes T cell priming by providing signal three (in addition to antigen recognition as signal one and costimulation as signal two) required for full activation of naive T cells, and enhances T cell survival by upregulating anti-apoptotic proteins including BCL-2 and BCL-XL. Type I interferon upregulates expression of major histocompatibility complex class I molecules on tumor cells, improving tumor cell recognition by CD8+ T cells. Type I interferon activates natural killer cells, which can directly kill tumor cells lacking adequate MHC class I expression and produce interferon-γ that further enhances dendritic cell and T cell functions [69,70,71,72,73]. Fourth, oncolytic virus infection induces production of T cell-recruiting chemokines including CXCL9 and CXCL10 (also known as interferon-γ-inducible protein-10 and monokine induced by interferon-γ, respectively), which bind CXCR3 receptors on activated T cells and guide T cell migration from circulation into tumor tissues [185]. Clinical studies have documented more than several fold increases in CD8+ T cell density within tumors following oncolytic virus administration, transforming immune-excluded cold tumors into T cell-inflamed hot tumors [186,187]. Fifth, and critically for combination therapy, type I interferon signaling drives upregulation of PD-L1 expression on tumor cells, tumor-associated macrophages, dendritic cells, and other cells within the tumor microenvironment through interferon regulatory factor signaling pathways [120]. Tumors that are PD-L1-negative at baseline and therefore unlikely to respond to checkpoint inhibitor monotherapy become PD-L1-positive following oncolytic virus treatment and are rendered sensitive to subsequent or concurrent checkpoint blockade [120,188].

Immune checkpoint inhibitors contribute complementary functions to the combination. Antibodies blocking the PD-1 receptor on T cells or the PD-L1 ligand on tumor cells prevent PD-1/PD-L1 interaction, thereby releasing the “brake” on T cell activation and preventing T cell exhaustion [1,2,4]. In the context of oncolytic virus-primed tumors with high PD-L1 expression and abundant tumor-infiltrating T cells, checkpoint blockade enhances the cytotoxic function of these T cells by maintaining their activation state, promoting interferon-γ production, granzyme and perforin expression, and cytolytic activity against tumor cells. Checkpoint inhibitors promote the formation of long-lived memory CD8+ T cells capable of recognizing and eliminating tumor cells upon re-exposure, providing the potential for durable complete responses that persist even after treatment discontinuation [1,2,4]. The systemic effects of checkpoint inhibitors complement the local effects of intratumorally administered oncolytic virus, enabling T cells primed in virus-injected lesions to traffic to and eliminate non-injected distant metastases, producing abscopal responses observed in subset patients depending on the specific combination [118,189,190].

**Figure 3:**
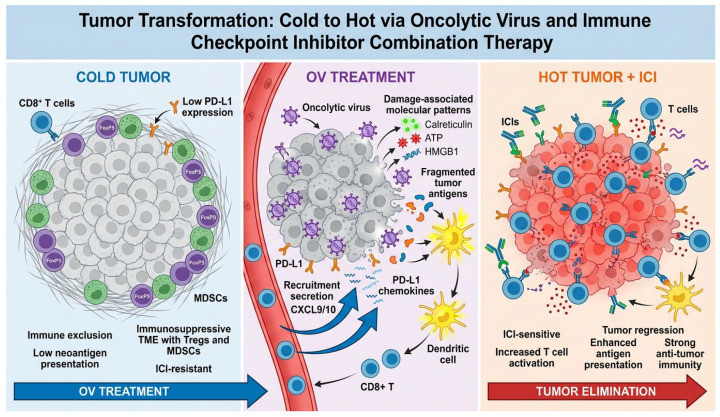
Molecular Mechanisms of Synergy Between Oncolytic Viruses and Immune Checkpoint Inhibitors.

The clinical outcome of combining oncolytic viruses with checkpoint inhibitors is transformation of the tumor phenotype from cold to hot. Pre-treatment tumor biopsies typically show immune-excluded phenotypes with minimal CD8+ T cell infiltration (fewer than 50 cells per square millimeter), absence of PD-L1 expression (less than 1% of tumor cells positive), lack of interferon-γ gene signatures, and immunosuppressive microenvironments dominated by regulatory T cells, myeloid-derived suppressor cells, and M2-polarized tumor-associated macrophages [8,9,120]. Post-treatment biopsies obtained 2–4 weeks after combination therapy initiation demonstrate dramatic transformation to hot tumor phenotypes characterized by dense CD8+ T cell infiltration, high PD-L1 expression and a strongly positive interferon-γ gene signature [26] (Figure 3).

Mechanisms underlying the synergistic anti-tumor efficacy of oncolytic virus (OV) and immune checkpoint inhibitor (ICI) combination therapy. (A) Pre-treatment: Immunologically “cold” tumors exhibit minimal CD8+ T cell infiltration, low PD-L1 expression, and immunosuppressive microenvironments dominated by regulatory T cells (Tregs) and myeloid-derived suppressor cells (MDSCs). Checkpoint inhibitor monotherapy response rate: 15–20%. (B) OV administration (Day 0–14): Intratumoral OV injection induces immunogenic cell death (ICD) with coordinated release of DAMPs (CRT at 6–12 h, ATP at 12–24 h, HMGB1 at 24–48 h) and viral PAMPs (dsRNA, 5′-triphosphate RNA), triggering type I IFN production (10-fold higher with RNA viruses vs. DNA viruses), dendritic cell (DC) recruitment and maturation (CD80/CD86 upregulation), tumor antigen cross-presentation, and de novo priming of tumor-specific CD8+ T cells. OV-mediated inflammation upregulates PD-L1 on tumor cells and increases CD8+ tumor-infiltrating lymphocyte (TIL) density, converting “cold” tumors to “hot” tumors. (C) ICI addition (Day 14–28): Anti-PD-1/PD-L1 antibodies block the adaptive immune resistance induced by OV-mediated PD-L1 upregulation, unleashing the expanded tumor-specific T cell population generated by OV-induced ICD. Anti-CTLA-4 antibodies enhance T cell priming in draining lymph nodes and deplete intratumoral Tregs through Fc-mediated ADCC. CXCL9/CXCL10 chemokine gradients (substantial increase) guide activated T cells from circulation into the tumor. (D) Synergistic outcome: Combined OV + ICI therapy achieves superior clinical efficacy (T-VEC + ipilimumab 39% ORR, T-VEC + pembrolizumab 62% ORR [phase Ib], CVA21 + pembrolizumab 47% ORR) versus monotherapy (ICI alone 20–45%, OV alone 15–30%), with abscopal responses in non-injected distant lesions mediated by circulating tumor-specific memory CD8+ T cells (observed in 38–64% of patients). Key predictive biomarkers include pre-treatment factors (baseline PD-L1 expression, IFN-γ gene signatures, viral receptor expression [ICAM-1 for CVA21, CVA11, CAR for CVB3], tumor mutational burden >10 mut/Mb) and early on-treatment changes increase in intratumoral CD8+ TILs, elevated serum HMGB1, interferon-stimulated gene upregulation, emergence of neoantigen-specific T cells by week 6).

### 5.3. Predictive Biomarkers for Response to Oncolytic Virus-Checkpoint Inhibitor Combinations

Identification of predictive biomarkers to guide patient selection for oncolytic virus-checkpoint inhibitor combinations represents a critical priority for maximizing clinical benefit while minimizing unnecessary treatment of patients unlikely to respond. Pre-treatment biomarkers assessed in tumor biopsies or blood samples before therapy initiation can identify patients with tumor and immune characteristics associated with a higher likelihood of response. PD-L1 expression measured by immunohistochemistry using validated assays (22C3, 28-8, SP263, or SP142 antibody clones) serves as a positive predictive biomarker, with PD-L1-high tumors (greater than 50% of cells positive or combined positive score greater than 10) demonstrating higher response rates to checkpoint inhibitor-containing regimens compared to PD-L1-negative tumors [191,192,193,194,195]. Interferon-g gene signatures quantified by RNA sequencing or NanoString analysis identify tumors with pre-existing T cell-inflamed phenotypes that respond favorably to immunotherapy [191,192,193,194,195]. Receptor expression for viral entry, specifically ICAM-1 for CVA21 and CVA11 or CAR for CVB3, measured by immunohistochemistry predicts viral infectivity and oncolytic activity, with high receptor expression (immunohistochemistry score 2+ or 3+) associated with superior outcomes [148,149,196]. Tumor mutational burden quantified by whole-exome sequencing or comprehensive genomic profiling identifies tumors with high numbers of somatic mutations (typically greater than 10 mutations per megabase) that generate abundant neoantigens capable of stimulating T cell responses [191,192,193,194,195]. Baseline tumor-infiltrating lymphocyte density assessed by immunohistochemistry or flow cytometry of dissociated tumor tissue, particularly CD8+ T cell density exceeding 100 cells per square millimeter, correlates with favorable outcomes [191,192,193,194,195]. Upregulation of interferon-stimulated genes including ISG15, MX1, and OAS1 measured by quantitative reverse transcription PCR in peripheral blood or tumor tissue indicates active type I interferon signaling and correlates with anti-tumor efficacy [68,69,70,71]. Although shown in NSCLC patients receiving combination therapy of ICI and chemotherapy, median PFS and OS rates were higher in patients with a ≥ 2-fold increase in plasma expression levels of CRT, one of DAMP molecules, than in those with a < 2-fold increase (PFS, 14.9 versus 6.0 months, hazard ratio (HR), 0.58; *p* = 0.17; OS, not reached versus 21.6 months, HR, 0.31, *p* = 0.02), suggesting that plasma CRT level monitoring has the potential to predict the efficacy [197]. Expansion of tumor antigen-specific T cells detected by tetramer staining, intracellular cytokine staining, or T cell receptor sequencing, with tumor-reactive T cells comprising 2–8% of circulating or tumor-infiltrating CD8+ T cells in responding patients compared to less than 0.5% in non-responders, directly measures successful T cell priming against tumor antigens [190,198,199].

Thus, integration of multiple biomarkers into composite signatures may improve predictive accuracy beyond individual biomarkers. Specifically, increased infiltration of CD8+ T cells and dendritic cells in the tumor microenvironment, elevated interferon-stimulated gene expression signatures, PD-L1 expression levels, and immune-related cytokine or chemokine profiles (including CXCL9, CXCL10, IFN-γ, and IL-12) have each been proposed as biomarkers that may enhance the prediction of oncolytic virus and immune checkpoint inhibitor combination efficacy. Although no universal biomarkers are definitively established, comprehensive immune profiling integrating tumor and circulating biomarker data can substantially improve prediction of treatment responses. A predictive model incorporating baseline PD-L1 expression, tumor mutational burden, interferon-γ gene signature, DAMP-related molecule expression and CD8+ T cell density may identify patients likely to achieve objective responses to the CVA21 plus pembrolizumab combination therapy. Such biomarker-driven approaches can enable precision medicine strategies that maximize the likelihood of benefit while avoiding unnecessary treatment toxicity and cost in patients unlikely to respond.

## 6. Discussion and Future Directions

### 6.1. Summary of Key Findings and Conclusions

This comprehensive review of oncolytic virotherapy and immunogenic cell death establishes several critical conclusions that advance our understanding of cancer immunotherapy mechanisms and guide future therapeutic development. First, oncolytic viruses induce robust immunogenic cell death characterized by four cardinal hallmarks occurring in precise spatiotemporal coordination: pre-apoptotic calreticulin exposure (6–12 h post-infection) serving as an “eat me” signal for dendritic cells, ATP secretion (12–24 h) functioning as a “find me” signal recruiting immune cells, HMGB1 release (24–48 h) providing TLR4-dependent dendritic cell maturation signals, and type I interferon production (6–24 h) directly activating dendritic cells and conditioning them for optimal antigen cross-presentation [30,31,32,33,34,35,132,133]. The defining feature distinguishing virus-induced ICD from chemotherapy- or radiation-induced ICD is the synergistic combination of damage-associated molecular patterns with viral pathogen-associated molecular patterns, creating danger signals that far exceed the immunogenicity of either DAMPs or PAMPs alone and resolving the fundamental challenge of stimulating immune responses against self-antigens including tumor-associated antigens [30,31,81]. Second, RNA virus platforms, particularly picornaviruses including coxsackieviruses, demonstrate superior immunogenicity compared to DNA virus platforms (Table 3) due to 10–100-fold higher type I interferon production resulting from minimal interferon evasion mechanisms. DNA viruses including Vaccinia, HSV-1, and Adenovirus encode multiple proteins (Vaccinia B18R, E3L, K3L; HSV-1 ICP34.5, ICP47; Adenovirus E1A, E3) that antagonize interferon production and signaling pathways, resulting in IFN-b production of only 5–50 U/mL compared to 300–2000 U/mL generated by RNA virus infections [68,86,87,90,91,92,93]. This differential in type I interferon production correlates with more robust dendritic cell activation, greater CD8+ T cell infiltration into tumors, superior tumor-specific T cell priming, and enhanced tumor rechallenge protection in preclinical models, with RNA virus-treated mice demonstrating 80–90% rejection of rechallenge tumors compared to 60–80% for DNA virus-treated mice [22,133,146,149,169,200]. Third, clinical efficacy of oncolytic viruses is maximized through rational combination with immune checkpoint inhibitors, with the highest reported objective response rate of 47% achieved using CVA21 plus pembrolizumab in melanoma patients in the CAPRA trial [141]. This represents approximately doubling of pembrolizumab monotherapy response rates and more than tripling of CVA21 monotherapy response rates, validating the mechanistic hypothesis that oncolytic viruses convert immunologically cold tumors lacking T cell infiltration into hot tumors densely infiltrated by activated tumor-specific CD8+ T cells that become responsive to checkpoint blockade. The molecular basis for this synergy involves oncolytic virus-mediated tumor lysis releasing antigens, induction of immunogenic cell death providing danger signals that activate dendritic cells, type I interferon-driven upregulation of PD-L1 expression sensitizing tumors to PD-1/PD-L1 blockade, and chemokine production recruiting T cells into previously immune-excluded tumors, complemented by checkpoint inhibitor-mediated prevention of T cell exhaustion and enhancement of T cell cytotoxic function (Figure 3) [25,26,120].

Fourth, platform-specific advantages and limitations inform optimal virus selection for particular clinical applications. Vaccinia virus offers very large transgene capacity (greater than 25 kb) enabling incorporation of multiple immunostimulatory genes but faces challenges of hepatotropism causing dose-limiting liver toxicity, high seroprevalence of neutralizing antibodies (30–50%) limiting systemic administration, and extensive interferon evasion reducing immunogenicity [82,83,84,85,95,96,97,98,99,100,101,102,105,201]. T-VEC benefits from established regulatory approval and clinical infrastructure, documented safety and efficacy in melanoma, and substantial clinical experience, but exhibits high seroprevalence (60–90%), moderate interferon evasion through ICP34.5 deletion, and limited efficacy as monotherapy (16% durable response rate) [20,90,91,92,117]. CVA21 demonstrates exceptional immunogenicity (10–100-fold higher IFN-I than DNA viruses), the robust clinical efficacy when combined with checkpoint inhibitors, and low seroprevalence (less than 20%), but has limited transgene capacity (less than 2 kb) restricting payload options [132,133,134,135,136,137]. CVA11 provides unique selectivity for NSCLC and malignant pleural mesothelioma through ICAM-1-dependent receptor tropism, maintained efficacy in chemotherapy-resistant colorectal carcinoma [146,147,148]. CVB3-miRT represents a paradigm-shifting safety engineering achievement, with microRNA-targeting providing significant cardiac attenuation while preserving complete oncolytic potency, full ICD-inducing capacity, and robust systemic anti-tumor immunity [149,169].

Fifth, predictive biomarkers enable precision medicine approaches to maximize clinical benefit. Pre-treatment biomarkers including PD-L1 expression, interferon-γ gene signatures, receptor expression (ICAM-1 for CVA21 and CVA11, CAR for CVB3), tumor mutational burden, and baseline T cell infiltration identify patients most likely to benefit [191,192,193,194,195,202].

### 6.2. Translational Barriers and Challenges Requiring Solutions

Despite remarkable preclinical and clinical advances, several translational barriers must be addressed to enable widespread clinical implementation of oncolytic virotherapy. Manufacturing and scalability represent substantial challenges, as clinical-grade production of replication-competent viruses at industrial scale requires doses of 10 to the 12th power to 10 to the 14th power viral particles per patient. This process necessitates specialized biosafety level 2 containment facilities with rigorous quality control systems capable of detecting adventitious agents and replication-competent contaminants at sensitivities below one infectious particle per million doses, and regulatory frameworks specifically designed for live biotherapeutics that differ fundamentally from traditional small molecule or biologic therapies [16,17,203]. RNA viruses present particular manufacturing challenges due to genetic instability during large-scale propagation in cell culture, with accumulation of defective interfering particles that reduce therapeutic viral titers and may alter biological properties [203].

Delivery routes and biodistribution optimization remain active areas of investigation. Intratumoral injection, currently the most common delivery route for clinical applications, limits treatment to accessible lesions and requires interventional radiology guidance for deep or visceral tumors, restricting applicability primarily to melanoma and head/neck cancers with easily accessible subcutaneous or mucosal lesions [203,204]. Intravenous delivery, which would enable treatment of widespread metastatic disease including visceral and bone metastases, faces substantial challenges including neutralizing antibodies that reduce viral biodistribution 10–100-fold through antibody-mediated clearance, rapid hepatic and splenic sequestration through Kupffer cell and marginal zone macrophage uptake, complement-mediated viral inactivation through antibody-independent and antibody-dependent pathways, and endothelial cell barriers preventing viral extravasation from blood vessels into tumor parenchyma [203,204]. Strategies under investigation to overcome these barriers include antibody-shielding approaches using polyethylene glycol conjugation that mask viral epitopes recognized by neutralizing antibodies, carrier cell delivery using mesenchymal stromal cells or T cells as “Trojan horses” that home to tumors and deliver virus while protecting it from antibody neutralization, pre-conditioning regimens using cyclophosphamide to deplete antibody-producing B cells and reduce neutralizing antibody titers, and combination with vascular-disrupting agents or vascular normalization therapies to enhance viral extravasation [203,204].

Host antiviral immunity represents a fundamental biological barrier that profoundly influences the efficacy of oncolytic virotherapy and must be carefully considered in therapeutic design. Upon oncolytic virus administration, the host innate immune system rapidly mounts antiviral responses through multiple complementary mechanisms. Pattern recognition receptors, including Toll-like receptors (TLR3, TLR7, TLR8, TLR9), RIG-I-like receptors, and cGAS-STING pathway components, detect viral nucleic acids and trigger production of type I and type III interferons that establish an antiviral state in surrounding uninfected cells, limiting viral spread within the tumor mass. Natural killer cells are rapidly recruited to sites of viral infection and eliminate virus-infected cells through perforin/granzyme-mediated cytotoxicity, further curtailing viral replication. Complement activation through both classical and alternative pathways contributes to viral neutralization and opsonization. While these innate antiviral responses limit the extent and duration of viral replication within tumors, they simultaneously contribute to the immunostimulatory effects that underlie oncolytic virotherapy efficacy: the very interferon responses and inflammatory cascades that restrict viral replication also activate dendritic cells, promote tumor antigen cross-presentation, and prime tumor-specific T cell responses. This dual role of antiviral immunity—simultaneously limiting direct viral oncolysis while amplifying anti-tumor immune responses—represents a central paradox in oncolytic virotherapy that must be carefully balanced through rational virus engineering and dosing strategies. Adaptive antiviral immunity further complicates the therapeutic landscape. Virus-specific CD8+ cytotoxic T cells and neutralizing antibodies generated following initial oncolytic virus exposure can rapidly clear virus upon repeat administration, potentially limiting the efficacy of multiple dosing cycles. The kinetics of this adaptive antiviral response vary by platform: neutralizing antibody titers typically peak 10–14 days after initial exposure for RNA viruses and 14–21 days for DNA viruses, with memory responses generating accelerated clearance upon subsequent exposures.

Pre-existing immunity resulting from natural infections or vaccination programs represents a particularly challenging barrier for DNA virus platforms. Vaccinia virus faces 30–50% seroprevalence in populations vaccinated during smallpox eradication programs, HSV-1 demonstrates 60–90% seroprevalence with substantial geographic and age-related variation, and adenovirus serotype 5 exhibits 50–90% seroprevalence in most human populations. Neutralizing antibodies can reduce viral tumor biodistribution by 10–100-fold following systemic administration and may limit efficacy even following intratumoral injection due to antibody penetration into tumor interstitium [90,93,94,101,102,103,104]. Alternative strategies include use of rare adenovirus serotypes with low seroprevalence (serotypes 11, 35, or 48), engineering of chimeric viruses incorporating capsid proteins from multiple serotypes to create novel antigen combinations not recognized by pre-existing antibodies, or selection of RNA virus platforms (CVA21, CVA11, CVB3-miR) with substantially lower seroprevalence (less than 20%) [93,94,132,133,149,200,205].

Tumor heterogeneity in receptor expression limits viral spread within tumor masses, as typically only 30–70% of tumor cells express sufficient levels of viral entry receptors (ICAM-1 for CVA21, CVA11, CAR for CVB3 and nectin-1 for HSV-1) to support productive infection [130,131,148,200,206]. Receptor-negative tumor cell subpopulations can escape direct viral oncolysis and may drive tumor progression following initial response [130,131,206]. Combination strategies may address this limitation, as immune checkpoint inhibitors enable virus-primed T cells to eliminate receptor-negative tumor cells through immune-mediated killing mechanisms, vascular-disrupting agents cause tumor ischemia affecting both infected and uninfected cells, and radiation therapy generates immunogenic cell death in uninfected cells while potentially upregulating viral entry receptors through interferon-γ production [130,131,204,206].

Immune-related adverse events increase in frequency when combining oncolytic viruses with checkpoint inhibitors compared to either monotherapy. The MASTERKEY-265 trial combining T-VEC with pembrolizumab reported grade 3–4 immune-related adverse events in 15% of patients, necessitating corticosteroid administration and treatment interruption in some cases [26,144]. The CAPRA trial combining CVA21 with pembrolizumab demonstrated 22% incidence of immune-related adverse events including hepatitis, thyroiditis, and colitis [141]. Biomarker-guided dose optimization, careful patient selection excluding those with pre-existing autoimmune conditions or significant comorbidities, and development of predictive biomarkers for immune toxicity may help mitigate these risks while maintaining therapeutic efficacy [26,144,190,199].

### 6.3. Future Directions and Next-Generation Strategies

Future development of oncolytic virotherapy will focus on next-generation engineering strategies, novel combination approaches, biomarker-driven precision medicine, and innovative clinical trial designs. Next-generation engineering encompasses multiple complementary strategies. Arming oncolytic viruses with immunostimulatory transgenes beyond GM-CSF may enhance anti-tumor immunity through multiple mechanisms: interleukin-12 drives Th1 polarization of CD4+ helper T cells and enhances cytotoxic T lymphocyte and natural killer cell functions [207], interleukin-15 promotes T cell and NK cell proliferation and survival while preventing activation-induced cell death [208], 4-1BBL provides costimulation to T cells through CD137 receptor engagement while simultaneously depleting intratumoral regulatory T cells [209], bispecific T cell engagers (BiTEs) recruit and activate T cells to tumor cells expressing specific antigens such as EGFR in lung and head/neck cancers [210], and PD-L1-blocking single-chain variable fragment antibodies expressed locally within tumors may achieve checkpoint blockade while avoiding systemic immune-related adverse events associated with systemic antibody administration [211].

Tumor microenvironment remodeling through expression of extracellular matrix-degrading enzymes may enhance viral spread and immune cell infiltration. Hyaluronidase expression (as in genetically engineered VCN-01 adenovirus and OVV-Hyal1 vaccinia virus) degrades hyaluronic acid polymers that create physical barriers to viral dissemination and T cell trafficking, particularly in pancreatic adenocarcinoma and other solid tumors [128,212]. Collagenase or matrix metalloproteinase expression degrades collagen-rich stroma, potentially improving viral spread in breast cancer, colorectal cancer, and other fibrotic tumors [204].

Expansion of microRNA-targeting strategies to additional virus platforms and target organs may enhance safety profiles. Liver-specific targeting using miR-122 target sequences can prevent hepatotoxicity, a dose-limiting toxicity for many systemically administered viruses. Brain-specific targeting with miR-124 sequences protects neurons from infection, critical for viruses with potential neurotropism including HSV-1 and measles virus. Hematopoietic cell targeting using miR-142-3p sequences prevents infection of lymphocytes, monocytes, and dendritic cells, which may be important for viruses that could potentially replicate in circulating immune cells [166,167,170,213,214]. Multitargeting incorporating sequences for multiple tissue-specific microRNAs may provide enhanced safety margins through redundant regulatory mechanisms [167,169,214].

Tumor-adaptive evolution through serial passage of oncolytic viruses in patient-derived tumor organoid cultures may generate variants with enhanced oncolytic potency, improved receptor binding affinity, or increased immunogenicity. This approach exploits viral genetic plasticity, particularly for RNA viruses with high mutation rates, to select for beneficial adaptations that enhance therapeutic performance [203,204,215,216].

Combination of oncolytic viruses with conventional chemotherapy represents another promising therapeutic strategy with a growing body of preclinical and clinical evidence. The rationale for this combination is multifaceted: chemotherapy can sensitize tumor cells to viral infection by attenuating intrinsic antiviral defense pathways, induce immunogenic cell death that synergizes with virus-induced ICD, reduce immunosuppressive cell populations (regulatory T cells, myeloid-derived suppressor cells) within the tumor microenvironment, and provide direct cytotoxic effects against virus-resistant tumor cell subpopulations. Clinically, the combination of H101 (an E1B-55K-deleted adenovirus) with cisplatin/5-fluorouracil chemotherapy demonstrated superior response rates compared to chemotherapy alone in head and neck squamous cell carcinoma, leading to regulatory approval in China in 2005 [123,124]. Preclinical studies have demonstrated that sequential administration of oxaliplatin followed by Coxsackievirus A11 produces greater tumor suppression than either therapy alone in chemotherapy-resistant colorectal cancer models, with chemotherapy appearing to attenuate intrinsic antiviral responses within tumor cells and thereby enhance viral replication [147]. The timing and sequencing of chemotherapy relative to oncolytic virus administration is critical: concurrent administration may impair viral replication due to cytotoxic effects on virus-infected cells, whereas sequential approaches—administering chemotherapy prior to or following oncolytic virus treatment—may optimize synergistic efficacy. Low-dose metronomic chemotherapy regimens that selectively deplete immunosuppressive cell populations while preserving effector T cell function may be particularly well-suited for combination with oncolytic viruses.

Novel combination strategies will exploit complementary mechanisms of action across therapeutic modalities. Triple combinations pairing oncolytic virus with checkpoint inhibitor and targeted therapy of BRAF/MEK inhibitors in BRAF-mutant melanoma may simultaneously address multiple tumor dependencies [217]. Oncolytic virus plus adoptive cell therapy combinations are particularly promising: oncolytic viruses reduce tumor burden, upregulate tumor antigen expression, induce inflammatory chemokine production, and create a permissive microenvironment for T cell function, while CAR-T cells or tumor-infiltrating lymphocytes eliminate virus-resistant tumor cell subpopulations and provide long-term immunological surveillance [218,219]. Sequential therapy optimizing the timing of different modalities may enhance efficacy: priming with oncolytic virus to generate tumor antigens and recruit immune cells, followed by checkpoint inhibitor administration to enhance T cell function when tumor-specific T cells are most abundant [25,26,93,220,221,222].

Biomarker-driven precision medicine will enable patient selection to enrich clinical trial populations for those most likely to benefit. Enrollment criteria based on ICAM-1 or CAR expression measured by immunohistochemistry (score 2+ or 3+), baseline PD-L1 expression (combined positive score greater than 10), interferon-γ gene signature (NanoString score in upper quartile), or tumor mutational burden (greater than 10 mutations per megabase) may increase response rates from 30–40% in unselected populations to 60–80% in biomarker-selected populations [148,191,192,193,194]. Adaptive enrichment designs that allow modification of enrollment criteria based on accumulating biomarker-outcome relationships may further optimize patient selection [93].

Clinical trial design innovations will accelerate development timelines and improve efficiency. Window-of-opportunity trials administering oncolytic virus in the neoadjuvant setting (2–6 weeks before planned surgery) enable on-treatment tumor biopsies to assess viral replication, immune infiltration, and pharmacodynamic biomarker changes, providing mechanistic insights that inform dose selection and combination strategies for subsequent trials [222,223]. Adaptive trial designs using response-adaptive randomization increase allocation of patients to superior treatment arms based on accumulating efficacy data, potentially reducing sample sizes required to demonstrate superiority while ensuring more patients receive effective therapy [93]. Basket trials enrolling patients based on biomarker status (ICAM-1-high or CAR-high tumors) rather than tumor histology may identify responsive tumor types that would be missed in traditional histology-based trials [143].

The field of oncolytic virotherapy is rapidly evolving, with several important recent publications advancing our understanding of OV-immune interactions. The references in this review have been updated to include recent contributions through early 2025. Regulatory pathway optimization through breakthrough therapy designation, accelerated approval mechanisms, and collaborative interactions with regulatory agencies may expedite clinical development. The FDA’s breakthrough therapy designation, granted to therapies demonstrating substantial improvement over existing options based on preliminary clinical evidence, provides intensive FDA guidance, organizational commitment to expedited development, and eligibility for rolling review and priority review [203]. Accelerated approval based on objective response rate or other surrogate endpoints in diseases with high unmet need, with post-approval confirmatory trials verifying clinical benefit, may provide earlier patient access while confirmatory data are being generated [19,203,224].

## 7. Conclusions and Future Outlook

Oncolytic viruses represent a transformative approach to cancer immunotherapy that addresses the fundamental limitation of immune checkpoint inhibitors: the inability to generate de novo immune responses in immunologically cold tumors lacking pre-existing T cell infiltration. By inducing robust immunogenic cell death characterized by spatiotemporally coordinated release of DMAPs and viral PAMPs, oncolytic viruses convert immunologically ignored tumors into inflamed lesions densely infiltrated by innate immune cells such as DCs and NK cells and tumor-associated antigen-specific CD8+ T cells. The synergy with immune checkpoint inhibitors has translated into unprecedented clinical efficacy, with coxsackievirus A21 combined with pembrolizumab achieving a 47% objective response rate in melanoma, representing notable efficacy exceeding either monotherapy.

RNA virus platforms, particularly picornaviruses including coxsackieviruses A21, A11, and B3, exhibit superior immunogenicity compared to DNA virus platforms due to 10–100-fold higher type I interferon production resulting from minimal interferon evasion mechanisms. Coxsackievirus A11’s unique ICAM-1-dependent receptor tropism provides exceptional selectivity for several solid tumors. Genetically manipulated innovation in microRNA-targeted coxsackievirus B3 demonstrates that rational safety engineering exploiting tissue-specific microRNA expression profiles can achieve robust reduction in organ toxicity while preserving complete oncolytic potency, full immunogenic cell death-inducing capacity, and robust systemic anti-tumor immunity, providing a roadmap for clinical translation of highly immunogenic RNA virus platforms.

Future success in oncolytic virotherapy requires addressing translational barriers including manufacturing scalability to produce the billions of viral particles required per patient dose, delivery optimization to enable systemic administration for widespread metastatic disease, strategies to circumvent pre-existing immunity that neutralizes viruses before they reach tumors, and approaches to overcome tumor heterogeneity that limits viral spread and enables emergence of resistant tumor cell populations. Combination strategies with conventional chemotherapy, which can sensitize tumor cells to viral infection and synergize with virus-induced immunogenic cell death, represent an additional avenue with growing clinical evidence. Next-generation strategies incorporating immunostimulatory transgenes (IL-12, IL-15, BiTEs, checkpoint-blocking antibodies), tumor microenvironment remodeling enzymes (hyaluronidase, collagenase), expanded microRNA-targeting for multi-organ safety (liver, brain, hematopoietic cells), and rational combination approaches with checkpoint inhibitors, molecularly targeted therapies, or adoptive cell therapy will further enhance therapeutic efficacy. Biomarker-driven patient selection enriching for individuals with high viral entry receptor expression, PD-L1 positivity, interferon-γ signatures, and high tumor mutational burden will maximize the likelihood of benefit while avoiding unnecessary treatment of patients unlikely to respond. Adaptive clinical trial designs, window-of-opportunity studies providing mechanistic insights, and collaborative regulatory pathways will accelerate development timelines and facilitate approval of effective new therapies.

As the field matures over the coming decade, oncolytic virotherapy is poised to transition from a niche experimental approach to a cornerstone of mainstream cancer immunotherapy, offering new hope to patients with immunologically cold tumors refractory to current checkpoint inhibitor-based treatments and potentially providing curative outcomes in diseases currently considered incurable.

Key Insights: (1) Synergistic efficacy: Combination therapies consistently demonstrate superior ORR compared to monotherapy (T-VEC alone 26% vs. T-VEC + pembrolizumab 62% [phase Ib]; CVA21 alone 28% vs. CVA21 + pembrolizumab 47%). (2) RNA virus advantage: CVA21 (Coxsackievirus) achieved 47% ORR when combined with pembrolizumab in the CAPRA trial, potentially reflecting superior IFN-I production and PD-L1 upregulation. (3) Abscopal effects: Responses observed in both injected and distant non-injected lesions across multiple trials, validating systemic immunity induction. (4) Complete responses: Substantial CR rates achievable with combinations (T-VEC + pembrolizumab 33% CR), representing potential cures. (5) Safety: Generally manageable toxicity profiles with flu-like symptoms most common; no unexpected synergistic toxicities. (6) Translational challenges: PHOCUS trial failure highlights importance of combination partner selection and predictive biomarkers. (7) Predictive biomarkers: Pre-treatment PD-L1, IFN-γ signatures, viral receptor expression, and early on-treatment TIL increases correlate with response. (8) Global development: Active trials across diverse tumor types including melanoma, bladder cancer, HCC, glioblastoma, and pancreatic cancer demonstrate broad platform applicability.

## Figures and Tables

**Table 1:** Comparative Immunogenic Cell Death Characteristics Across Oncolytic Virus Platforms.

ICD Characteristic	Vaccinia Virus	HSV-1 (T-VEC)	Adenovirus	CVA21	CVB3-miRT	Measurement Method
Genome Type	dsDNA, 190 kb	dsDNA, 150 kb	dsDNA, 36 kb	ssRNA(+), 7.4 kb	ssRNA(+), 7.4 kb	—
Replication Site	Cytoplasm	Nucleus	Nucleus	Cytoplasm	Cytoplasm	—
Replication Kinetics	24–48 h	18–24 h	18–30 h	12–24 h	12–24 h	Time-lapse microscopy
CRT Exposure	++ (12–18 h)	++ (10–16 h)	++ (12–18 h)	+++ (6–12 h)	+++ (6–12 h)	Flow cytometry, IF
ATP Secretion	++ (18–24 h)	++ (16–22 h)	++ (18–24 h)	+++ (12–18 h)	+++ (12–18 h)	Luciferase bioluminescence
HMGB1 Release	++ (24–48 h)	++ (20–36 h)	++ (24–36 h)	+++ (18–30 h)	+++ (18–30 h)	ELISA, Western blot
Type I IFN	+ (Moderate)	++ (Mod-High)	++ (Mod-High)	+++ (Very High)	+++ (Very High)	qPCR, ELISA
PAMP Recognition	cGAS-STING, TLR2	cGAS-STING, TLR9	cGAS-STING	RIG-I, MDA5, TLR7/8	RIG-I, MDA5, TLR7/8	Reporter assays
IFN Evasion	B18R, E3L, K3L	ICP34.5 (del in T-VEC)	E1A, E3 proteins	Minimal	Minimal	Functional assays
DC Maturation	++	++	++	+++	+++	CD80/CD86/MHC-II flow
CD8+ TIL Increase	++	+++	++	+++	+++	IHC, flow cytometry
ORR (Monotherapy)	10–20%	26%	15–25%	28.1%	Pre-clinical	Clinical trials
ORR (+ICI)	N/A (PHOCUS: +sorafenib [TKI], terminated)	39–62% (+ipi/pem)	33–47% (+pembro)	47% (+pembro)	Not yet tested	Clinical trials
Safety Concerns	Pre-existing immunity	Generally well-tolerated	Liver tropism	Generally well-tolerated	Cardiac: miR-targeting	AE monitoring

Legend: ORR = objective response rate; ICI = immune checkpoint inhibitor; ipi = ipilimumab; pembro = pembrolizumab; ; TIL = tumor-infiltrating lymphocytes; DC = dendritic cell; IFN = interferon; PAMP = pathogen-associated molecular pattern; CRT = calreticulin; HMGB1 = high-mobility group box 1; ATP = adenosine triphosphate. Scoring: + = moderate; ++ = strong; +++ = very strong. RNA viruses (CVA21, CVB3) demonstrate earlier and more robust ICD characteristics compared to DNA viruses, particularly in type I IFN production and kinetics of DAMP release, correlating with superior immune activation and clinical efficacy when combined with checkpoint inhibitors.

**Table 3:** Comparative Advantages and Disadvantages of DNA Versus RNA Oncolytic Virus Platforms.

Feature	DNA Virus Platforms (Vaccinia, HSV-1, Adenovirus)	RNA Virus Platforms (CVA21, CVA11, CVB3-miRT)
Genome	dsDNA; 36–190 kb	ssRNA(+); 7.4 kb
Replication site	Nucleus (HSV-1, Adeno) or Cytoplasm (Vaccinia)	Cytoplasm
Transgene capacity	Large (7–35 kb depending on vector generation; Vaccinia >25 kb)	Very limited (<2 kb); minimal engineering space
IFN evasion mechanisms	Extensive: B18R, E3L, K3L (Vaccinia); ICP34.5, ICP47 (HSV-1); E1A, E3 (Adeno)	Minimal: lack dedicated IFN antagonist genes
Type I IFN production	Low to moderate (5–50 U/mL IFN-β)	High (300–2000 U/mL IFN-β); >10 fold higher than DNA viruses
ICD induction kinetics	Slower onset (CRT 10–18 h, ATP 16–24 h, HMGB1 20–48 h)	Faster onset (CRT 6–12 h, ATP 12–18 h, HMGB1 18–30 h)
Pre-existing immunity	High: Vaccinia 30–50%, HSV-1 60–90%, Ad5 50–90%	Low: CVA21 <20%, CVB3 <25%
Manufacturing	Well-established large-scale production; genetically stable	Genetic instability during propagation; defective interfering particles
Clinical development	Most advanced: T-VEC FDA-approved (2015); G47Δ Japan-approved (2021); H101 China-approved (2005)	Advancing: CVA21 Phase Ib/II (CAPRA, CALM); CVA11 preclinical; CVB3-miRT preclinical
Receptor tropism	Broad; HSV-1: nectin-1; Adeno: CAR	ICAM-1 (CVA21, CVA11); CAR (CVB3); selectively overexpressed on tumors
Key advantages	Large payload; established manufacturing; regulatory precedent; engineerability	Superior IFN-I induction; faster replication; low seroprevalence; potent ICD; safety engineering (miRT)
Key disadvantages	IFN evasion limits immunogenicity; high seroprevalence; hepatotropism; PHOCUS failure	Limited transgene capacity; genetic instability; no regulatory approvals yet; cardiac risk (CVB3 wt)

Legend: dsDNA = double-stranded DNA; ssRNA(+) = positive-sense single-stranded RNA; IFN = interferon; ICD = immunogenic cell death; CRT = calreticulin; HMGB1 = high-mobility group box 1; Ad5 = adenovirus serotype 5; miRT = microRNA-targeted. Data compiled from references cited in the main text.

## Data Availability

No new data were created or analyzed in this study.
